# Enhancing microalgal lipid accumulation for biofuel production

**DOI:** 10.3389/fmicb.2022.1024441

**Published:** 2022-10-10

**Authors:** Zhi Zhu, Jing Sun, Yun Fa, Xufeng Liu, Peter Lindblad

**Affiliations:** ^1^The Key Laboratory of Biotechnology for Medicinal Plants of Jiangsu Province, School of Life Sciences, Jiangsu Normal University, Xuzhou, China; ^2^State Key Laboratory of Biocatalysis and Enzyme Engineering, School of Life Sciences, Hubei University, Wuhan, China; ^3^CAS Key Laboratory of Bio-Based Materials, Qingdao Institute of Bioenergy and Bioprocess Technology, Chinese Academy of Sciences, Qingdao, China; ^4^Microbial Chemistry, Department of Chemistry-Ångström Laboratory, Uppsala University, Uppsala, Sweden

**Keywords:** microalgae, lipid enhancement, biofuel production, metabolic engineering, process optimization

## Abstract

Microalgae have high lipid accumulation capacity, high growth rate and high photosynthetic efficiency which are considered as one of the most promising alternative sustainable feedstocks for producing lipid-based biofuels. However, commercialization feasibility of microalgal biofuel production is still conditioned to the high production cost. Enhancement of lipid accumulation in microalgae play a significant role in boosting the economics of biofuel production based on microalgal lipid. The major challenge of enhancing microalgal lipid accumulation lies in overcoming the trade-off between microalgal cell growth and lipid accumulation. Substantial approaches including genetic modifications of microalgal strains by metabolic engineering and process regulations of microalgae cultivation by integrating multiple optimization strategies widely applied in industrial microbiology have been investigated. In the present review, we critically discuss recent trends in the application of multiple molecular strategies to construct high performance microalgal strains by metabolic engineering and synergistic strategies of process optimization and stress operation to enhance microalgal lipid accumulation for biofuel production. Additionally, this review aims to emphasize the opportunities and challenges regarding scaled application of the strategic integration and its viability to make microalgal biofuel production a commercial reality in the near future.

## Introduction

Biofuel is a form of energy which captures solar energy as chemical energy in the bonds of biologically produced materials (Srivastava et al., 2020). As one of the most important study aspects in exploitation and application of the renewable energy, biofuel plays a significant role in dealing with the increasing demand of energy and the deteriorating environmental pollution problems (Medipally et al., 2015; Ong et al., 2020; Peng et al., 2020). Compared with others, lipid-based biofuels have been attracting extensive attention due to the higher energy density, better infrastructure compatibility and greater application flexibility (Wang et al., 2022).

Unicellular microalgae are photoautotrophic organisms which grow like photosynthetic plants while lacking the complex cell structures of higher plants (Slade and Bauen, 2013). Microalgae have been considered as one of the most promising alternative sustainable feedstocks for producing lipid-based biofuels due to their higher lipid accumulation capacity, higher growth rate and higher photosynthetic efficiency compared to the traditional plants (Chisti, 2007; Chu, 2017; Anto et al., 2020; Wang et al., 2020). In addition, microalgae provide proteins that can be used as feed source for animals (Amorim et al., 2021). Some of them can also produce high value biologically active compounds like some antioxidant pigments (Markou and Nerantzis, 2013). Microalgae have been displayed greater sustainable and commercial advantages as feedstock for biofuels production (Harun et al., 2010; Saranya and Shanthakumar, 2021).

Microalgae could offer great prospect for biofuel exploitation. However, the process is still not carbon neutral and commercially viable because of the high production cost (Behera et al., 2021; Brar et al., 2021). Enhancing microalgal lipid accumulation could improve the economic feasibility of the biofuel production. Several recent reviews have summarized genetic and metabolic engineering approaches and/or cultivation regulating strategies for enhancing microalgal lipid accumulation or productivity, but a very few discussed these strategies all together for achieving high lipid production with more focus on the trade-off between microalgal cell growth and lipid accumulation (Chu, 2017; Sun et al., 2019; Khan and Fu, 2020; Shokravi et al., 2020; Brar et al., 2021).

The focus of this review is thus to highlight the advancements and emerging approaches towards achieving enhancement of microalgal lipid accumulation for biofuel production on the basis of the trade-off between microalgal cell growth and lipid accumulation. The scope of present work covers genetic manipulations of microalgal strains and optimizations of microalgal cultivation systems, along with their challenges.

## Construction of high-efficient lipid producing microalgae

**Figure 1:**
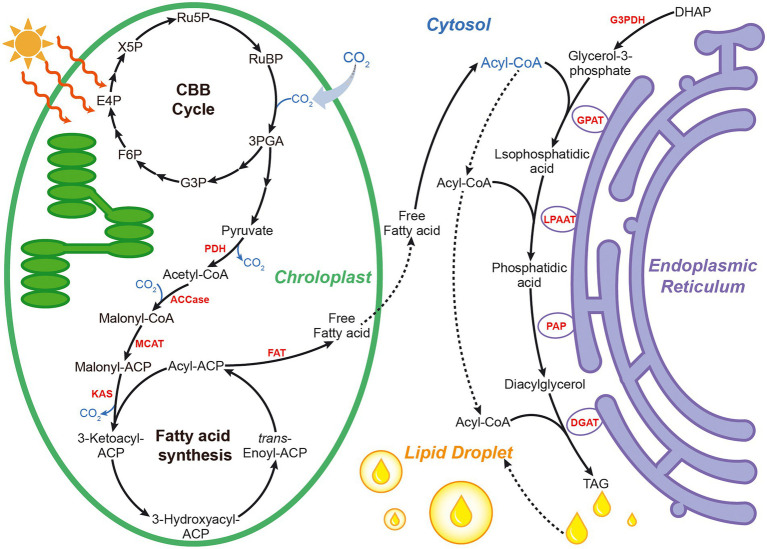
Schematic overview of metabolic pathways involved in lipid synthesis in microalgae. 3PGA, 3-phosphoglycerate; ACCase, acetyl-CoA carboxylase; DGAT, diacylglycerol acyltransferase; DHAP, dihydroxyacetone phosphate; E4P, erythrose-4-phosphate; F6P, fructose-6-phosphate; FAT, fatty acyl-ACP thioesterase; G3P, glyceraldehyde-3-phosphate; G3PDH, gycerol-3-phosphate dehydrogenase; GPAT, glycerol-3-phosphate acyltransferase; KAS, 3-ketoacyl-ACP synthase; LPAAT, lysophosphatidic acid acyltransferase; MCAT, malonyl-CoA: acyl carrier protein transacylase; PAP, phosphatidic acid phosphatase; PDH, pyruvate dehydrogenase complex; Ru5P, ribulose-5-phosphate; RuBP, ribulose-1,5-bisphosphate; TAG, triacylglycerols; X5P, xylulose-5-phosphate.

There are native biological routes for biofuel molecules in some of the natural strains of microalgae. Figure 1 gives a schematic overview of lipid metabolic pathways in microalgae. Microalgal lipid metabolic pathways are mainly based on fatty acid *de novo* biosynthesis pathway and triacylglycerol (TAG) synthesis route. Fatty acid *de novo* biosynthesis pathway in microalgae occurs in chloroplast. As primary substrate of fatty acid *de novo* biosynthesis, acetyl-CoA is carboxylated to malonyl-CoA generating saturated fatty acids, which go through further desaturation and elongation forming unsaturated fatty acids catalyzed by complex fatty acid synthases (FAS; Moffett et al., 2020; Behera et al., 2021). TAG synthesis pathway in endoplasmic reticulum has been proposed to be composed of three sequential transfers of acyl group from acyl-CoA to glycerol-3-phosphat (Brar et al., 2021; Mulgund, 2022). The natural production was generally low, restricting the industrialized production and commercialized development of the microalgal biofuels. Metabolic engineering is one of the most important research fields of biotechnology innovation, which helps to modify metabolic pathways to trigger the productions of the target biofuel metabolites.

### Metabolic engineering of fatty acid *de novo* biosynthesis pathway

Malonyl-CoA: acyl carrier protein transacylase (MCAT) is responsible for the conversion of malonyl-CoA to malonyl acyl carrier protein, which is the first committed step of fatty acid biosynthesis, followed by the reduction-dehydration-reduction reaction cycle. Overexpression of MCAT was shown to increase the accumulation of the fatty acid (Lei et al., 2012; Tian et al., 2013). For example, the MCAT overexpressed *Schizochytrium* obtained 110.5 g/l total lipid in the fed-batch cultivation, which was 39.6% higher than that in cells of the wild strain. In addition, the production of polyunsaturated fatty acid was also increased when overexpressing MCAT (Li et al., 2018). Overexpression of MCAT in oleaginous microalga *Nannochloropsis oceanica* elevated the lipid content to 42.9% of the dry cell weight, leading to 36.0% higher content than that in cells of the wild strain (Chen et al., 2017).

Acetyl-CoA carboxylase (ACCase) is responsible for the conversion of acetyl-CoA to malonyl-CoA, making malonyl-CoA to enter the fatty acid biosynthesis pathway (Figure 1). Many studies have shown that upregulation of ACCase improve the biosynthesis of the fatty acid (Sun et al., 2019). Increasing ACCase expression by genetic engineering to overproduce fatty-acid based biofuels has been widely employed in model microorganisms such as *Escherichia coli* and *Saccharomyces cerevisiae* (Das et al., 2020). Gomma et al. were the first to report overexpressed ACCase for improving fatty acid biosynthesis in microalgae. The total fatty acid production of ACCase overexpressed mutant was increased by 60% compared to in cells of the wild type (Gomma et al., 2015).

Glucose-6-phosphate dehydrogenase (G6PD) is involved in the biosynthesis of NADPH in pentose phosphate pathway, which plays an important role in maintaining the reducing power and redox homeostasis. Xue et al. constructed a G6PD overexpressed mutant of *Phaeodactylum tricornutum* in which both the transcript abundance and enzyme activity of G6PD were increased as a result of enhancement of NADPH (Xue et al., 2017). The lipid content reached 55.7% of dry cell weight, 2.7-fold higher than that in cells of the wild type (Xue et al., 2017). By enhancing the reducing power supply, overexpression of G6PD in microalgae can significantly improve the lipid accumulation, illustrating that G6PD may be a promising metabolic engineering target for efficient microalgal lipid production. Overexpression of malic enzyme was also proved playing a significant role in enhancing neutral lipid production of *P. tricornutum* through the additional supply of NADPH (Zhu et al., 2018).

### Metabolic engineering of triacylglycerol synthesis pathway

In microalgae, glycerol-3-phosphate dehydrogenase (G3PDH) catalyzes the conversion of dihydroxyacetone phosphate (DHAP) to glycerol-3-phosphate in the cytosol, followed by glycerol-3-phosphate acyltransferase (GPAT) which catalyzes the conversion of glycerol-3-phosphate to lysophosphatidic acid, lysophosphatidic acid acyltransferase (LPAAT) the conversion of lysophosphatidic acid to phosphatidic acid, phosphatidic acid phosphatase (PAP) the conversion of phosphatidic acid to diacylglycerol, and finally diacylglycerol acyltransferase (DGAT) the conversion of diacylglycerol to triacylglycerol (TAG) in the endoplasmic reticulum (Korkhovoy and Blume, 2013; Figure 1). Hsieh et al. adopted a multiple gene expression strategy to elevate the lipid accumulation of microalgae (Hsieh et al., 2012). The coordinated overexpression of G3PDH, GPAT, LPAAT, PAP, and DGAT from *Saccharomyces cerevisiae* and/or *Yarrowia lipolytica* increased the lipid production of *Chlorella minutissima* 2-fold compared to in cells of the wild type (Hsieh et al., 2012). Wang et al. multi-overexpressed homologous GPAT and LPAAT in *Phaeodactylum tricornutum*, leading to 2.3-fold higher TAG content (with nitrogen stress condition) than that in cells of the wild strain (Wang et al., 2018). Compared with other TAG biosynthesis-related genes, the genes encoding GPAT and DGAT may be more effective targets for harnessing lipid accumulation in the TAG biosynthesis. Zou et al. also provided a multiple gene expression platform for manipulation of complex metabolic nodes. Coordinated expression of homologous GPAT and DGAT increased the lipid content of *Phaeodactylum tricornutum* by 2.6-fold than that of wild type (Zou et al., 2018). Zulu et al. also heterologously co-expressed DGAT from yeast and oleosin (lipid droplet stabilizing protein) from plant in *Phaeodactylum tricornutum* resulting in a 3.6-fold increased TAG content compared to in cells of the wild strain (Zulu et al., 2017). With respect to conventional single gene construction, Niu et al. reported that overexpression of homologous GPAT alone made *P. tricornutum* to produce twise as much neutral lipids compared to in wild type cells (Niu et al., 2016). Chen et al. constructed a genetically engineered strain of *Scenedesmus obliquus* harboring a DGAT gene from *Chlamydomonas reinhardtii*, which was successfully cultured in a 40 l tubular photobioreactor. The lipid content of this recombinant strain reached 12.3% of dry cell weight, 128% higher than in cell of the wild strain (Chen et al., 2016). Collectively, metabolic engineering of key enzymes in the TAG biosynthesis pathway may be a promising strategy for microalgal lipid accumulation as required.

In addition to metabolic engineering for increasing the quantity of microalgal lipids, it is also rational to improve the quality of the producing lipids. Some efforts have been made on improving the lipids quality in terms of manipulating the degree of fatty acid unsaturation and the length of fatty acid carbon chain (Norashikin et al., 2018; Haslam et al., 2020; Wang et al., 2021).

### Metabolic engineering of competitor pathways

Knockout or knockdown of key enzymes of competing pathways to allow more carbon flux to be channeled toward the target products is widely considered as an effective strategy to improve the production of the target products. Microalgae accumulate starch (carbohydrate) and together with lipids they are the two primary carbon storage metabolites under stressful conditions (Takeshita et al., 2014; Li T. T. et al., 2015). The metabolism of starch and lipids are highly related, and glyceraldehyde-3-phosphate (G3P) is their common precursor (Ran et al., 2019). Blocking this competitive starch photosynthetic pathway channeling the carbon flux toward lipid biosynthesis may represent an effective strategy to overproduce the target lipid. For instance, a *Chlamydomonas* starchless mutant was found to accumulate 10-fold more cellular TAG compared to that in cells of wild type when the cultures were transferred to a high light intensity and nitrogen-less medium (Li et al., 2010). de Jaeger et al. reported that the total fatty acid productivity of a *Scenedesmus obliquus* starchless mutant increased 41% compared to in wild type cells and the TAG yield reached 49.4% of the dry cell weight under nitrogen deficiency stress condition (de Jaeger et al., 2014).

In microalgae, tricarboxylic acid (TCA) cycle and *de novo* fatty acid biosynthesis pathway are also two competitive pathways of carbon storage, sharing the common mid-metabolite phosphoenolpyruvate (PEP). Phosphoenolpyruvate carboxylase (PEPC) is responsible for the conversion of PEP to oxaloacetate (OAA), making OAA to enter into TCA cycle. Disruption of TCA cycle may be an effective approach for carbon partitioning toward lipid accumulation. A PEPC down- regulated strain of *Chlamydomonas reinhardtii* was developed in which the maximal lipid content and productivity increased by 74.4 and 94.2%, respectively, compared in wild type cells (Kao and Ng, 2017).

Inhibition of competitive lipid catabolism pathway is another practical metabolic engineering strategy targeting overall lipid accumulation. For example, Trentacoste et al. developed transgenic strains of *Thalassiosira pseudonana* through targeted knocking down of multifunctional lipase/phospholipase/lysophosphatidic acyltransferase, resulting in 3.3-fold higher lipid yield than that of wild type cells at exponential phase and 4.1-fold higher lipid yield than under silicon deficiency stress condition without affecting cell growth (Trentacoste et al., 2013).

## Optimization of nutrient conditions

**Figure 2:**
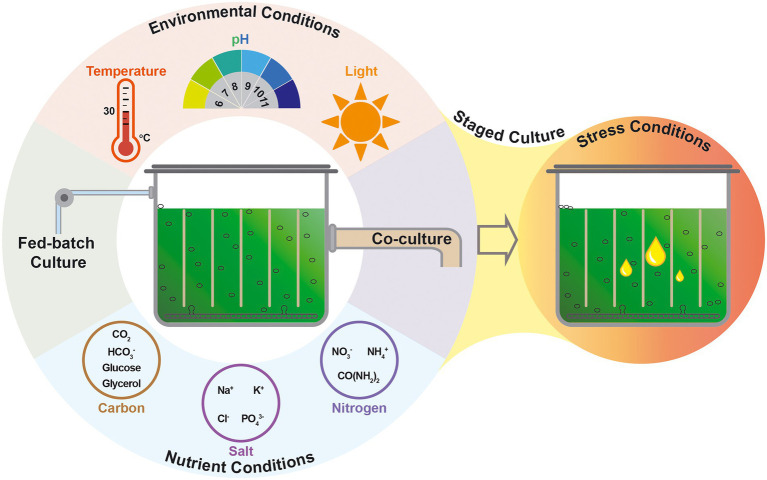
Systematic strategies of cultivation process tailoring microalgae for enhanced lipid production.

It is important to obtain high-efficient microalgae for industrialized biofuel production. To realize the full potential of the high-efficient microalgae, it is essential to optimize the process of their growth and the products production (Figure 2). Generally, the guideline of optimizing nutrient conditions is to investigate nutrients uptake from the medium and the metabolic routes of the nutrients, then to confirm the effect of different nutrient conditions on microalgal cell growth and metabolites distribution, before finally to further optimize the nutrient conditions for improving the production of biomass and/or target products (Du et al., 2006; Ramirez-Lopez et al., 2016). Targeting the overall biofuel production based on microalgae, the major challenge of the optimization of culture conditions, as well as the genetic modification of the microalgal host strain, lies in the trade-off between microalgal cell growth and lipid accumulation (Singh et al., 2016). The content and the supplying mode of the key ingredient of nutrients, such as carbon source or nitrogen source, play significant roles in the cell growth and the lipid accumulation are summarized in Figure 2.

### Carbon source

In photoautotrophic cultivation mode, microalgae utilize CO_2_ and NaHCO_3_ as inorganic carbon source to produce organic metabolites through photosynthesis. While in heterotrophic or mixotrophic mode, some microalgae assimilate organic molecules such as glucose or glycerol as carbon source and /or energy (Markou et al., 2014; Saranya and Shanthakumar, 2021). Tang et al. reported that the photosynthetic lipid content of both of *Scenedesmus obliquus* and *Chlorella pyrenoidosa* increased with increasing levels of environmental CO_2_ (Tang et al., 2011). The maximum lipid content of *S. obliquus* reached 24.4% of dry cell weight under 50% CO_2_ condition, 61% higher than that of 0.03% CO_2_ condition (simulating ambient atmosphere). The maximum lipid content of *C. pyrenoidosa* reached 26.8% of dry cell weight under 50% CO_2_ condition, 28% higher than that of 0.03% CO_2_ condition (Tang et al., 2011). Compared with photoautotrophic cultivation, the maximum lipid content of *Chlorella sorokiniana* increased 2.4-fold with 20 g/l glucose under heterotrophic cultivation and 3.9-fold with 8 g/l glucose under mixotrophic cultivation conditions (Li et al., 2014). Laraib et al. obtained 137.43 ± 13.3 mg/l/d of biomass productivity and 39% of lipid content during the mixotrophic cultivation of *Chlorella vulgaris* when molasses utilized as additional carbon source, respectively 1.5-fold and 2-fold higher than that of photoautotrophic cultivation (Laraib et al., 2021). Pang et al. introduced sodium gluconate using as an unconventional organic carbon source for the mixotrophic cultivation of *Haematococcus pluvialis* (Pang et al., 2019b). Yang et al. utilized sodium acetate to counter the growth inhibition of *Chlamydomonas reinhardtii* by nutrient deficiency, the lipid production increased by 93% under same nutrient deficiency condition (Yang et al., 2018). Li et al. reported that the lipid contents of *Chlorella* sp. strains were significantly higher under 1–30% CO_2_ concentrations after adaptive evolution than those of the original strain (Li D. J. et al., 2015). Taking together, regardless of photoautotrophic, heterotrophic, or mixotrophic cultivation, optimum carbon concentrations are required to enhance the lipid accumulation.

### Nitrogen source

Nitrogen is one of the nutrients most directly influencing the microalgal lipid accumulation (Shin et al., 2018; Feng et al., 2020). It may be provided in different forms, such as nitrate, urea or ammonium. However, different forms of nitrogen sources are specific for the growth and the lipid accumulation of microalgae. (Li et al., 2008; Gonzalez-Garcinuno et al., 2014; Feng et al., 2020). Of all the optimizing strategies of nitrogen source, nitrogen deficiency stress appears to be the most effective approach to stimulate the lipid accumulation in microalgae (Wu et al., 2013; Gonzalez-Garcinuno et al., 2014; Feng et al., 2020). For instance, Arguelles and Martinez-Goss found that there was an increasing trend in the lipid production of both of *Chlorolobion* sp. and *Chlorella* sp. under nitrogen limited culture condition in the range from 1.5 g/l to 0.375 g/l NaNO_3_ (Arguelles and Martinez-Goss, 2021). The lipid productivity of *Chlorolobion* sp. and *Chlorella* sp. reached 227.84 and 151.14 mg/l/d at 0.375 g/l NaNO_3_ concentration condition, respectively, 2.9-fold and 1.9-fold higher compared to at 1.5 g/l NaNO_3_ growth condition (Arguelles and Martinez-Goss, 2021). Feng et al. demonstrated that *Chlorella* cells showed a 48.65% lipid content of dry cell weight under nitrogen limitation condition, 62% higher than that under nitrogen sufficient condition (Feng et al., 2020). Gao et al. investigated the effects of three types of nutrients (nitrogen, phosphate and iron) starvation stress on the lipid production of *Chaetoceros muelleri* and *Dunaliella salina*, and observed the highest lipid contents when the cells cultured under a nitrogen deprivation condition (Gao et al., 2013).

It is widely acknowledged that by nitrogen deficiency stress, microalgae cell division decrease, channeling the lipid biosynthesis pathway toward neutral lipids rather than membrane lipids (Goncalves et al., 2016; Feng et al., 2020). As such, nitrogen limitation may also cause the production of biomass to decrease, which affects the further accumulation of lipids. A combination strategy of nitrogen deficiency stress and carbon enrichment might be an alternative option, supplying carbon source for fatty acid production along with stimulating the lipid biosynthesis pathway. Bharte and Desai revealed that the lipid contents of *Chlorella minutissima* and *Chlorella pyrenoidosa* were increased to 24 and 23% under the condition of nitrogen deprivation combined with acetate as additional carbon source, respectively 4 and 5.4% higher than those of a single nitrogen deprivation condition (Bharte and Desai, 2019). Zhu and Huang also reported that high glucose combined with low nitrogen could increase the lipid content of *Chlorella sorokiniana* under heterotrophic cultivation condition (Zhu and Huang, 2017).

## Optimization of environmental conditions

The production of biomass and the accumulation of lipid of microalgae are coordinated with both nutrient conditions and environmental conditions (Rehman et al., 2022). Optimizing environmental parameters and regulating the environmental conditions to improve the synthesis of microalgal lipid is an efficient strategy to increase the microalgal lipid productivity and reduce the cost of microalgal biofuels production (Figure 2).

### Temperature

Temperature is one of the most essential environmental parameters during the microalgal lipid production process, different temperatures not only change the nature of the nutrients but also affect the activities of various key enzymes in the metabolic process of the microalgal cells. Therefore, the effect of temperature on microalgal lipid accumulation is the comprehensive performance of various factors, and strain specific. In Wu et al.’s study, a decrease from 32.9 to 29.6% in the lipid content of a *Monoraphidium* strain was observed when the environmental temperature increased from 25 to 35°C (Wu et al., 2013). While in Converti et al.’s study, an increase in the temperature from 25 to 30°C led to a decrease in the lipid content of *C. vulgaris* from 14.71 to 5.90%, in contrast to that, the lipid content of *Nannochloropsis oculata* increased from 7.90 to 14.92% when the temperature increased from 25 to 30°C (Converti et al., 2009). Temperature plays more significant role in the growth of microalgae, however, to maximize lipid production of microalgae, it is essential to obtain robust and sufficient biomass as the foundation before the stage of lipid accumulation.

### pH

Environmental pH value is a very significant comprehensive indicator of microalgal metabolic activities under certain circumstances, and has influences on the dynamic forms and the relative concentration of inorganic carbon source in the culture medium (Azov, 1982). Therefore, pH plays an important role in the cell growth and lipid accumulation of microalgae. Moheimani investigated the effects of pH value on the lipid productivities of *Tetraselmis suecica* and *Chlorella* sp. (Moheimani, 2013). The maximum lipid productivity of *T. suecica* reached 92 ± 13.1 mg/l/d when the pH value was kept at 7.5, and that of *Chlorella* sp. 99 ± 17.2 mg/l/d when the pH was kept at 7.0 (Moheimani, 2013). According to Zhang et al. the maximal lipid production (167.5 mg/l) of *Chlorella* sp. was also observed at initial pH of 7.0 (Zhang et al., 2014). Qiu et al. evaluated the effects of different pH on the lipid production of a strain of *Chlorella sorokiniana* (Qiu et al., 2017). By adjusting the pH value through feeding CO_2_, an optimal pH for lipid accumulation was observed at 6.0, and the cetane numbers of biodiesel produced at pH 6.5, 7.0 and 7.5 fulfill the diesel standard (Qiu et al., 2017).

### Light

Microalgae utilize light as driving force to obtain chemical energy during photoautotrophic cultivation (Blair et al., 2014). The availability of light, which could be achieved through multiple manipulations, is essential for cell growth and lipid accumulation of microalgae. For instance, Liu et al. investigated the effects of different light intensities on the lipid accumulation of *Scenedesmus* sp. and all the maximum biomass production, lipid content and neutral lipid content were obtained when the strain of *Scenedesmus* was cultivated at 400 μmol photons m^−2^ s^−1^ light intensity and limited nitrogen content (Liu et al., 2012). He et al. investigated the effects of different fluctuating light intensities on the lipid productivity of oleaginous microalgae and observed the maximum lipid productivities and neutral lipid contents when the microalgae were cultured under high fluctuating light intensity (He et al., 2015). Jung et al. found that green LED produced the highest lipid content while blue LED led to the highest biomass (Jung et al., 2019). By contrast, Sánchez-Saavedra et al. found that the lipid content was much higher under blue light under exponential growth phase compared with white, green and yellow light (Sanchez-Saavedra et al., 2020). Feng et al. investigated the effects of different light paths in a photobioreactor on the microalgal lipid content (Feng et al., 2020). Compared with higher light paths, the maximum lipid content was achieved when a strain of *Chlorella* was cultured at 5 cm light path of the flat plate photobioreactor (Feng et al., 2020). Lima et al. found that low-frequency flashing light can improve the fatty acid productivity up to 3 times compared to that of the continuous light (Lima et al., 2021). Recently, nanoparticles have been emerging as spotlight for enhancing the microalgal lipid production by boosting the light conversion (Khanra et al., 2020; Vargas-Estrada et al., 2020).

## Optimization of cultivation system

### Staged cultivation

Cell growth of microalgae provides a solid foundation for the efficient biosynthesis of microalgal lipid. Optimization and regulation of nutritional and environmental conditions should not only supply superior conditions for cell growth but also satisfy the stress demand for lipid accumulation. In this sense, staged cultivation system emerged as a potential culture method applied for microalgal biofuel production since it is able to overcome the trade-off between the lipid accumulation with cell growth. In the staged cultivation system, commonly a two-stage approach, a nutrient-rich culture condition is provided in the first stage for maximum biomass, whereas stress culture conditions are applied for improving the microalgal lipid accumulation in the second stage (Figure 2).

Nutrient starvation, especially nitrogen starvation, has been regarded as the most reliable stress condition for stimulating the lipid production of microalgae in the staged cultivation system. Rai et al. employed nitrogen starvation condition during the two-stage cultivation of *Chlorella* sp. and the neutral lipid content increased to 50.43% of dry cell weight after 5-days of nitrogen starvation stress (Rai et al., 2017). Ghidossi et al. applied higher carbon to nitrogen ratio as nitrogen starvation stress condition in the second stage during two-stage culture process of *Chlorella protothecoides* and the lipid content reached 58% of dry cell weight with a lipid productivity of 2–5 fold higher compared with previously reported (Ghidossi et al., 2017). Other than nitrogen starvation, staged cultivation systems based on phosphorus starvation or silica starvation may also enhance microalgal lipid accumulation (Alvarez-Diaz et al., 2014; Smith et al., 2016). However, the effects of these nutrients starvation on the lipid accumulation seem to be more species dependent.

Considering the extra cost created in the transformation of microalgal biomass from nutrient-rich medium into nutrient-limitation medium when the nutrient starvation strategy is adopted in the lipid accumulation stage, nutrient starvation based staged cultivation system is less economically viable for the commercial application. In this regard, staged cultivation system based on environmental stress condition might be more feasible applied in large scale for microalgal lipid production. Ra et al. carried out a two-stage cultivation based on light stress condition and observed a maximal lipid content of 56% of dry cell weight in *Nannochloropsis oculata* under green light stress for 2 days in the second culture stage (Ra et al., 2016). By direct feeding of NaCl into the culture medium of *Scenedesmus obtusus* in the second stage, the lipid contents obtained under different concentrations of NaCl were all higher than that of single stage cultivation without the saline stress condition (Xia et al., 2013). A maximal lipid content of 47.7% of dry cell weight was obtained after 8 days stress of 20 g/l NaCl. When the same saline stress strategy applying at larger scale of 140 l, the lipid content of *S. obtusus* reached 42.1% of dry cell weight (Xia et al., 2013), illustrating that the staged cultivation system based on saline stress is not only effective but also practical for enhancing the microalgal lipid production. Higher salinity stress condition will induce higher lipid production, however, the tolerance of microalgae to high salinity is finite, and to high salinity could bring the growth inhibition even in the stage of lipid accumulation. To alleviate the biomass inhibition by high salinity stress for further enhancing the lipid accumulation, strategies for obtaining improved tolerance performance of microalgae to higher salinity stress have been proposed. For instance, Ho et al. administered a salinity gradient strategy by a stepwise sea salt addition during the two-stage cultivation of marine *Chlamydomonas*. The maximum lipid content reached 59.4% of dry cell weight after the optimal salinity gradient mode operated for 5 days, when 95% of nitrogen was consumed (Ho et al., 2014). In addition, the lipid productivity was also much higher compared with that of other staged cultivation system (Ho et al., 2014). This synergistic operation combining salinity gradient stress with nitrogen starvation stress develops positive impact on the trade-off between lipid production with lipid productivity during the staged cultivation process of microalgae. Furthermore, for more complex staged culture system based on multi-stress condition, Wang et al. integrated glucose fed-batch operation in the cell growth stage and hyperosmotic combined with nitrogen starvation stress condition in the lipid accumulation stage during the two-stage cultivation process of *Chlorella protothecoides* (Wang et al., 2017). The lipid content, lipid yield on glucose and lipid productivity obtained in the multi-stress two-stage fed-batch culture system increased 1.92, 1.79 and 1.60-fold compared to a no stress single stage fed-batch culture system, respectively (Wang et al., 2017).

Applying staged cultivation system based on nitrogen starvation stress is one of the most reliable strategies for enhancing the microalgal lipid production. While environmental stress operation is practically more feasible utilizing in large scale for industrialization. Consequently, integrating environmental stress operation when the culture system of microalgae is under nitrogen starvation stress condition may significantly enhance the microalgal lipid production during the large-scale staged cultivation process. The optimization and regulation of the entire stress-integrated staged cultivation process are of great importance for the commercialization of microalgal lipid production.

### Heterotrophic and mixotrophic cultivation

Some species of microalgae have the ability to grow heterotrophically or mixotrophically, displaying considerable metabolic diversity and flexibility (Morales-Sanchez et al., 2017; Pang et al., 2019a). Heterotrophic cultivation refers to the culture mode in which microalgae exclusively use organic substrates as both carbon and energy source (Yin et al., 2020). Liu et al. compared the effects of culture modes on the cell growth and lipid yield of *Chlorella* sp., heterotrophic culture mode was regarded as the optimal strategy for the accumulation of microalgal lipid (Liu et al., 2019). Ghidossi et al. investigated the effect of C/N ratio on the microalgal growth and lipid productivity of *Chlorella protothecoides* under heterotrophic cultivation, the maximum lipid productivity and microalgal biomass reached 16.7 g/l/d and 255 g/l, respectively, when the different C/N ratios were combined during the heterotrophic cultivation process (Ghidossi et al., 2017). Heterotrophic cultivation mode could easily manipulate the C/N ratio for optimizing the microalgal cell growth and the lipid accumulation. However, it worth noting that the economic viability of the heterotrophic cultivation utilizing the organic substrates.

Mixotrophic cultivation is a special mode in which microalgae can metabolize both organic and inorganic carbon source simultaneously under solar energy (Wang et al., 2014). Mixotrophic cultivation may be an ideal culture mode for large-scale microalgal lipid production because of its combined advantages of synergism of photoautotrophic and heterotrophic cultivation (Pang et al., 2019a; Patel et al., 2020). Heredia-Arroyo et al. studied the mixotrophic cultivations of *Chlorella vulgaris* by different organic carbon source at different concentrations (Heredia-Arroyo et al., 2011). 0.19 g/l of lipid production was obtained when 4 g/l glucose was applied as the initial organic carbon source, 77% higher than that of photoautotrophic culture condition (Heredia-Arroyo et al., 2011). Gao et al. regulated the ratio of organic carbon and nitrogen source during the mixotrophic cultivation of *Chlorella* sp. G-9 and observed a 36.5% of lipid content of dry cell weight and a lipid productivity of 32.6 mg/l/d, 13-fold higher than that in photoautotrophic culture condition (Gao et al., 2019). Based on the mixotrophic culture mode, Xue et al. constructed a co-culture system of *Spirulina platensis* and yeast *Rhodotorula glutinis* (Xue et al., 2010). Four hundred sixty-seven milligram per liter of total lipid production was obtained, 2-fold higher than the sum of that of the two single systems under the same mixotrophic culture condition (Xue et al., 2010). Qin et al. constructed a co-culture system of *Chlorella pyrenoidosa* and yeast *Yarrowia lipolytica* using glycerol as organic carbon source during the mixotrophic cultivation (Qin et al., 2019). The maximum lipid production reached 0.77 g/l, 2.85-fold higher than that of single system of *Y. lipolytica* and 3.53-fold higher than that of the single system of *C. pyrenoidosa* (Qin et al., 2019).

Compared with the improved lipid production or productivity, mixotrophic cultivation has a much more pronounced influence on the microalgae cell growth. Consequently, the enhancement of lipid content of microalgae seems to be less obvious or barely noticeable. It might be favorable to integrate the mixotrophic cultivation mode with staged cultivation system based on stress condition to accumulate higher microalgal lipid with good performance in terms of overcoming the trade-off between the lipid accumulation with the cell growth.

## Concluding remarks and future perspectives

Microalgae have been drawing tremendous attention as a promising emerging feedstock for the production of lipid-based biofuels. Economical and commercial application of microalgal biofuel production is subject to the enhancement of lipid accumulation on the basis of overcoming the conflicts between microalgal cell growth and lipid accumulation. Extensive efforts have been made on improving microalgal lipid accumulation including genetic modifications of microalgal strains by metabolic engineering and process regulations of microalgae cultivation by integrating multiple optimization strategies widely applied in industrial microbiology (Table 1). In future, in-depth understanding of the microalgal lipid metabolic network is essential for the construction of high-performance microalgal strains through metabolic engineering and molecular modification. Emerging omics techniques, including metabolomics, proteomics, and lipidomics, have been exhibiting great potential for further identifying and understanding of the microalgal lipid biosynthetic pathways by cooperating with genetic engineering (Arora et al., 2018; Rawat et al., 2021). Systematic optimization strategies integrating various biomass improvement strategies with nutrient and environmental stress operation during the staged cultivation mode should be developed for the maximization of microalgal lipid accumulation. More assessment of these synergistic strategies applying in large-scale microalgal lipid production with economic feasibility are still required. These advancements for enhancing microalgal lipid accumulation are certainly making biofuel production based on microalgae a reality for commercial application in the near future.

**Table 1:** Selected species of microalgae accumulate lipid for biofuel production.

Microalgal species	Lipid content	References
*Phaeodactylum tricornutum*	60.6% dry cell weight (DCW)	Jung et al. (2019)
*Chlamydomonas* sp.	59.4% of DCW	Ho et al. (2014)
*Chlorella protothecoides*	58% of DCW	Ghidossi et al. (2017)
*Phaeodactylum tricornutum*	57.5% of DCW	Zou et al. (2018)
*Nannochloropsis oculata*	56% of DCW	Ra et al. (2016)
*Phaeodactylum tricornutum*	55.7% of DCW	Xue et al. (2017)
*Chlorella* sp.	53.5% of DCW	Feng et al. (2020)
*Scenedesmus obliquus*	49.4% of DCW	de Jaeger et al. (2014)
*Scenedesmus obtusus*	47.7% of DCW	Xia et al. (2013)
*Nannochloropsis oceanica*	42.9% of DCW	Chen et al. (2017)
*Chlorella vulgaris*	39% of DCW	Laraib et al. (2021)
*Chlorella sorokiniana*	32% of DCW	Zhu and Huang (2017)

## Author contributions

ZZ and XL conceived the outline and drafted the manuscript. JS and YF revised the manuscript. PL made major revisions of the manuscript. All authors contributed to the article and approved the submitted version.

## Funding

This research was funded by Open Project Funding of the State Key Laboratory of Biocatalysis and Enzyme Engineering (Grant Number SKLBEE2020015), CAS Key Laboratory of Bio-based Materials, Chinese Academy of Sciences (Grant Number BMF-2020-08), Natural Science Foundation of the Jiangsu Higher Education Institutions of China (Grant Number 20KJB180012), and the China Scholarship Council (Scholarship Number 202108320126).

## Conflict of interest

The authors declare that the research was conducted in the absence of any commercial or financial relationships that could be construed as a potential conflict of interest.

## Publisher’s note

All claims expressed in this article are solely those of the authors and do not necessarily represent those of their affiliated organizations, or those of the publisher, the editors and the reviewers. Any product that may be evaluated in this article, or claim that may be made by its manufacturer, is not guaranteed or endorsed by the publisher.
